# Tumor microenvironment deconvolution identifies cell-type-independent aberrant DNA methylation and gene expression in prostate cancer

**DOI:** 10.1186/s13148-023-01609-3

**Published:** 2024-01-03

**Authors:** Samuel R. Reynolds, Ze Zhang, Lucas A. Salas, Brock C. Christensen

**Affiliations:** 1 Department of Epidemiology, Geisel School of Medicine at Dartmouth, Lebanon, NH USA; 2 Department of Molecular and Systems Biology, Geisel School of Medicine at Dartmouth, Lebanon, NH USA

**Keywords:** Prostate cancer, Tumor microenvironment, Deconvolution, Epigenome-wide association study, DNA methylation, Differential expression analysis

## Abstract

**Background:**

Among men, prostate cancer (PCa) is the second most common cancer and the second leading cause of cancer death. Etiologic factors associated with both prostate carcinogenesis and somatic alterations in tumors are incompletely understood. While genetic variants associated with PCa have been identified, epigenetic alterations in PCa are relatively understudied. To date, DNA methylation (DNAm) and gene expression (GE) in PCa have been investigated; however, these studies did not correct for cell-type proportions of the tumor microenvironment (TME), which could confound results.

**Methods:**

The data (GSE183040) consisted of DNAm and GE data from both tumor and adjacent non-tumor prostate tissue of 56 patients who underwent radical prostatectomies prior to any treatment. This study builds upon previous studies that examined methylation patterns and GE in PCa patients by using a novel tumor deconvolution approach to identify and correct for cell-type proportions of the TME in its epigenome-wide association study (EWAS) and differential expression analysis (DEA).

**Results:**

The inclusion of cell-type proportions in EWASs and DEAs reduced the scope of significant alterations associated with PCa. We identified 2,093 significantly differentially methylated CpGs (DMC), and 51 genes associated with PCa, including *PCA3*, *SPINK1*, and *AMACR*.

**Conclusions:**

This work illustrates the importance of correcting for cell types of the TME when performing EWASs and DEAs on PCa samples, and establishes a more confounding-adverse methodology. We identified a more tumor-cell-specific set of altered genes and epigenetic marks that can be further investigated as potential biomarkers of disease or potential therapeutic targets.

**Supplementary Information:**

The online version contains supplementary material available at 10.1186/s13148-023-01609-3.

## Background

This study aims to investigate cellular composition in the TME of prostate cancer patients to investigate epigenetic and gene expression alterations associated with prostate carcinogenesis. The prostate gland is a walnut-sized gland below the bladder, surrounding the urethra, and it is crucial to reproduction through its role in seminal fluid production and secretion. Prostate cancer is the second most common cancer in men behind nonmelanoma skin cancer—it is estimated that there will be 288,300 new cases and 34,700 deaths due to prostate cancer in the United States in 2023 [1]. One in every eight men will be diagnosed with prostate cancer during their lifetime, meaning more than 1.2 million new cases will be diagnosed each year worldwide [1]. Also, one in every 41 men will die of prostate cancer, making it the second leading cause of male cancer death behind lung cancer [2]. Most prostate cancer begins in the peripheral zone, or back of the prostate, and grows slowly. While the tumor remains in the prostate, it does not pose a serious health risk, and if detected early, minimally invasive procedures can successfully treat the disease. Often, prostate cancer identified in its early stages is monitored and only treated upon exhibiting evidence of metastatic potential. The five-year survival rate of patients whose cancer has spread outside the prostate is 31%, whereas the five-year survival rate of patients whose cancer was localized to the prostate is nearly 100% [3]. Prostate cancer is a complex disease, and several genetic alterations in prostate tumors have been identified. Frequent genetic alterations include fusions of *TMPRSS2* with ETS family genes, amplification of the *MYC* oncogene, deletion and/or mutation of *PTEN*and *TP53*, and mutation of the androgen receptor gene have been identified as contributors to prostate cancer [4].

Epigenetic modifications to DNA methylation are well known to occur in cancers, including prostate cancer [5]. DNA methylation helps regulate gene expression by either recruiting proteins, such as histone modifying or chromatin remodeling enzymes, that contribute to gene repression or by directly preventing the binding of transcription factors (TF) to DNA. During development and cellular differentiation, cell-type-specific methylation patterns are established and contribute to regulating cell-specific gene and protein expression patterns. DNA methylation patterns have been employed to estimate tumor purity, and subsequent differential methylation analyses have corrected for tumor purity [6]. Additionally, transcriptomic alterations in prostate cancer have been studied using differential expression analyses [5, 7, 8] including those with correction for immune cell types of the tumor microenvironment [9].

Epigenetic and transcriptomic alterations in prostate cancer have been studied, but previous analyses have solely focused on tumor purity or immune cell-type proportions. They have not addressed the variation in immune, tumor, and angiogenic cell-type proportions within the tumor microenvironment, which could be a crucial potential confounding factor across subjects. Determining the TME cell-type proportions can be accomplished through cell-type deconvolution, separating mixed signals into their individual parts. In a tumor sample, beyond tumor cells, there are numerous cell types, such as healthy prostate epithelium, blood and lymphatic vessels, and immune cell types, each of which has distinct epigenetic and gene expression patterns. Cell-type-specific epigenetic patterns can differentiate a given cell type from the others and calculate its proportion in the sample. Multiple TME deconvolution tools have been developed, including *CIBERSORT* [10] which uses RNA-seq data, and *MethylCIBERSORT* [11], *MethylResolver* [12], and Hierarchical Tumor Immune Microenvironment Epigenetic Deconvolution (*HiTIMED*) [13] which use DNA methylation data. *MethylCIBERSORT* and *MethylResolver* both trained their models on cancer cell lines instead of primary cancer cells and can only deconvolve ten and twelve immune cell types, respectively. Additionally, *MethylResolver* uses a universal standard reference for tumor purity estimation in all tumor types instead of using organ-specific epithelial cell-type DNA methylation signatures. Lastly, *CIBERSORT* and *methylCIBERSORT* force the deconvolution of every immune cell type, even if they are not present in the sample. In comparison, our *HiTIMED* method offers deconvolution of more cell types and has higher accuracy than similar methods. *HiTIMED* uses primary cancer cells and tissue-specific methylation signatures to deconvolve seventeen different tumor, immune, and angiogenic cell types more accurately with a hierarchical approach and does not force the deconvolution of cell types if they are not present. Here we used *HiTIMED* to investigate epigenetic and transcriptomic alterations in prostate cancer. We show the importance of adjusting for cell type in epigenome-wide association studies (EWAS) and identify altered DNA methylation and disease-specific gene expression.

## Results

The prostate cancer data used in this study consisted of 56 patients who underwent radical prostatectomies prior to any treatment and included tumor, adjacent non-tumor prostate tissues (GSE183040). Study subject demographics and clinical characteristics are provided in Table 1.

**Table 1:** Study population demographic and disease characteristics

Characteristics	Cases
Total subjects	56
Total samples	168
Age, mean (range)	63.1 (57.5–68.6)
Type of sample (Tumor, Benign, Buffy Coat)	(56, 56, 56)
Race (White, Black, Unknown)	(114, 21, 33)
Preoperative PSA Level, mean (range)	7.1 (1.2–13.0)
Pathological stages, n (%)	
Stage 2 (T2a, T2b, T2c)	33 (58.9)
Stage 3 (T3a, T3b)	23 (41.1)
Gleason scores, n (%)	
Primary pattern of 3 (3 + 3 T4, 3 + 4, 3 + 4 T5)	39 (69.6)
Primary pattern of 4 and 5 (4 + 3, 4 + 3 T5, 4 + 4, 4 + 5, 5 + 4, 5 + 5 T4)	17 (30.4)

**Fig. 1:**
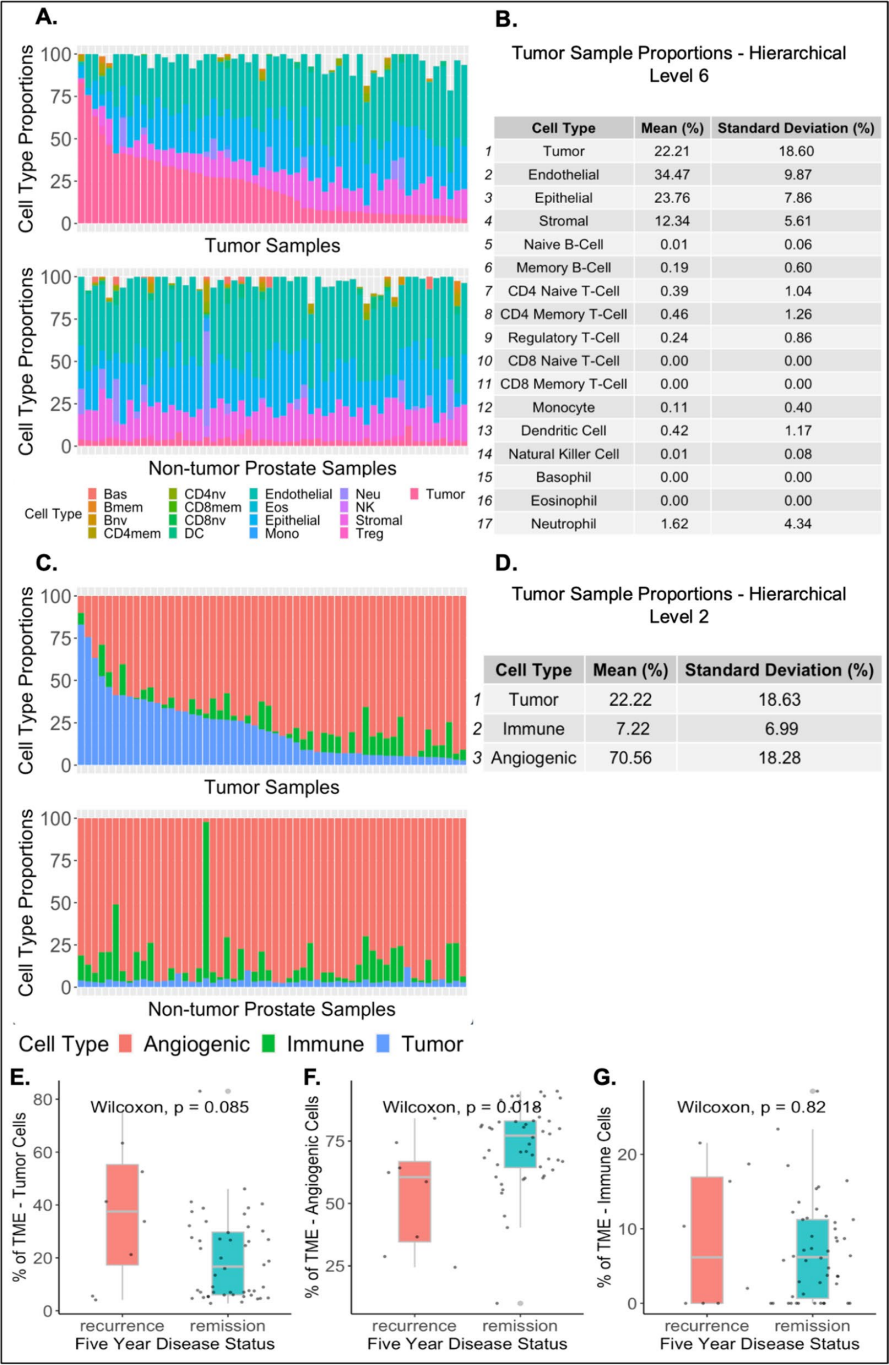
Prostate cancer tumor deconvolution and association of cell type with five-year remission and recurrence status. **A** Monocytes, basophils, eosinophils, neutrophils, naïve B, memory B, CD4 naïve T, CD4 memory T, regulatory T, CD8 naïve T, CD8 memory T, dendritic cell, natural killer, endothelial, epithelial, stromal, and tumor cell-type proportions of tumor and non-tumor prostate tissue ordered on tumor cell percentage of the tumor sample. **B** Mean cell-type proportions of the prostate tumor samples at *HiTIMED* hierarchical level six. **C** Angiogenic (endothelial, epithelial, and stromal cells), immune, and tumor cell-type proportions of tumor and non-tumor prostate tissue ordered on tumor cell percentage of the tumor sample. **D** Mean cell-type proportions of the prostate tumor samples at *HiTIMED* hierarchical level two. Five-year remission and recurrence status based on the tumor **E**, angiogenic **F**, and immune **G** cell-type proportions of the TME

Cell-type proportions in the tumor microenvironment (TME) were quantified using genome-scale DNA methylation data measured with the Illumina Infinium MethylationEPIC platform from the tumor and adjacent non-tumor prostate tissue for all subjects using the *HiTIMED* package in R. The prostate TME was deconvolved at the second HiTIMED hierarchical layer (Fig. 1C). The second layer returns tumor, immune (naïve B, memory B, CD4 naïve T, CD4 memory T, regulatory T, CD8 naïve T, CD8 memory T, monocyte, dendritic cell, natural killer, basophil, eosinophil, and neutrophil cells), and angiogenic (endothelial, epithelial, and stromal cells) cell-type proportions. Mean cell-type proportions of the second deconvolution layer for tumor and healthy prostate tissues are reported in Fig. 1D.

Univariate comparisons of the three higher-level cell-type categories (tumor, immune, and angiogenic) from HiTIMED hierarchical level two between patients who experience remission versus recurrence of their tumor are shown in Fig. 1. A higher proportion of tumor cells in the TME at the time of radical proctectomy approached significance (*P* = 0.085) for patients who experienced recurrence (Fig. 1E). Immune cell-type proportions are not significantly associated with the recurrence of a tumor (*P* = 0.82) (Fig. 1F), while decreased proportions of angiogenic cells in the TME were associated with recurrence (*P* = 0.018) (Fig. 1G).

**Fig. 2:**
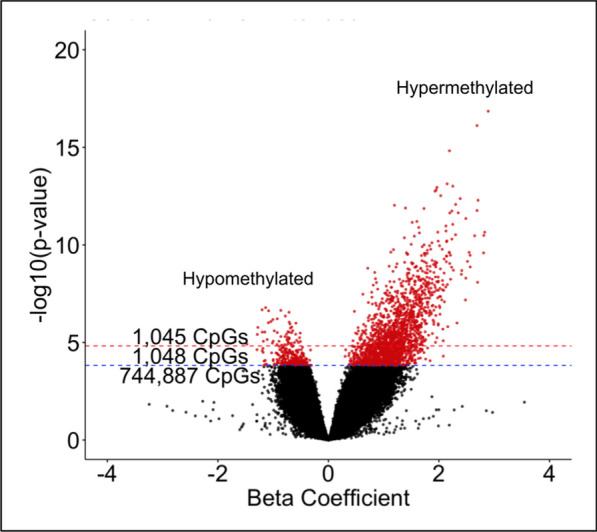
TME correction in EWAS identifies 2093 tumor-specific CpGs when comparing tumor versus non-tumor groups. Epigenome-wide association study of prostate cancer and matched non-tumor normal prostate tissue adjusted for patient age, race, Gleason score, pathological stage, and preoperative PSA levels, immune and angiogenic cell-type proportions from HiTIMED hierarchical level two, and blocked on patient ID. Each point represents a CpG site; in total, 746,980 CpG sites are shown, and those with an FDR *Q*-value < 0.05 are shown in red (2093 CpGs). FDR *Q*-value < 0.05 is shown above the blue line, and FDR *Q*-value < 0.01 is shown above the red line

Although previous studies have investigated alterations in the epigenome of prostate tumors including correction for the TME, published work to date has only included corrections for tumor purity or immune cell types of the TME only, and not a more complete correction using tumor, immune, and angiogenic cell types. *HiTIMED* does not force the prediction of TME cell types. Therefore, there were missing cell-type proportions in the hierarchical level 6 (Fig. 1A and B), which is why hierarchical level 2 was chosen to correct for the TME, as it did not contain missing values. To assess the extent of potential confounding by cell type, three different epigenome-wide association study (EWAS) sets of models were fitted using paired tumor non-tumor prostate tissue samples from 56 patients. For all three EWASs, the Gleason score was grouped into those with a primary pattern of three and those with a primary pattern of four or five, and the pathological stage was grouped into those in stage two and those in stage three. In the first EWAS, models testing DNA methylation between prostate tumor and non-tumor tissue adjusting for age and race resulted in 363,454 significantly differentially methylated CpG sites (FDR *Q*-value < 0.05, Additional file 1: Fig. S1A). The second EWAS adjustment for age, race, Gleason score, pathological stage, and preoperative PSA levels resulted in 361,330 significantly differentially methylated CpG sites (FDR *Q*-value < 0.05, Additional file 1: Fig. S1B). The third EWAS adjusted for variables in the second EWAS and immune and angiogenic cell-type proportions from HiTIMED, resulting in 2093 significantly differentially methylated CpG sites (FDR *Q*-value < 0.05, Additional file 1: Fig. S1C, Fig. 2). Of the 2,093 CpG sites, 285 were hypomethylated, and 1808 were hypermethylated in tumors relative to normal.

**Fig. 3:**
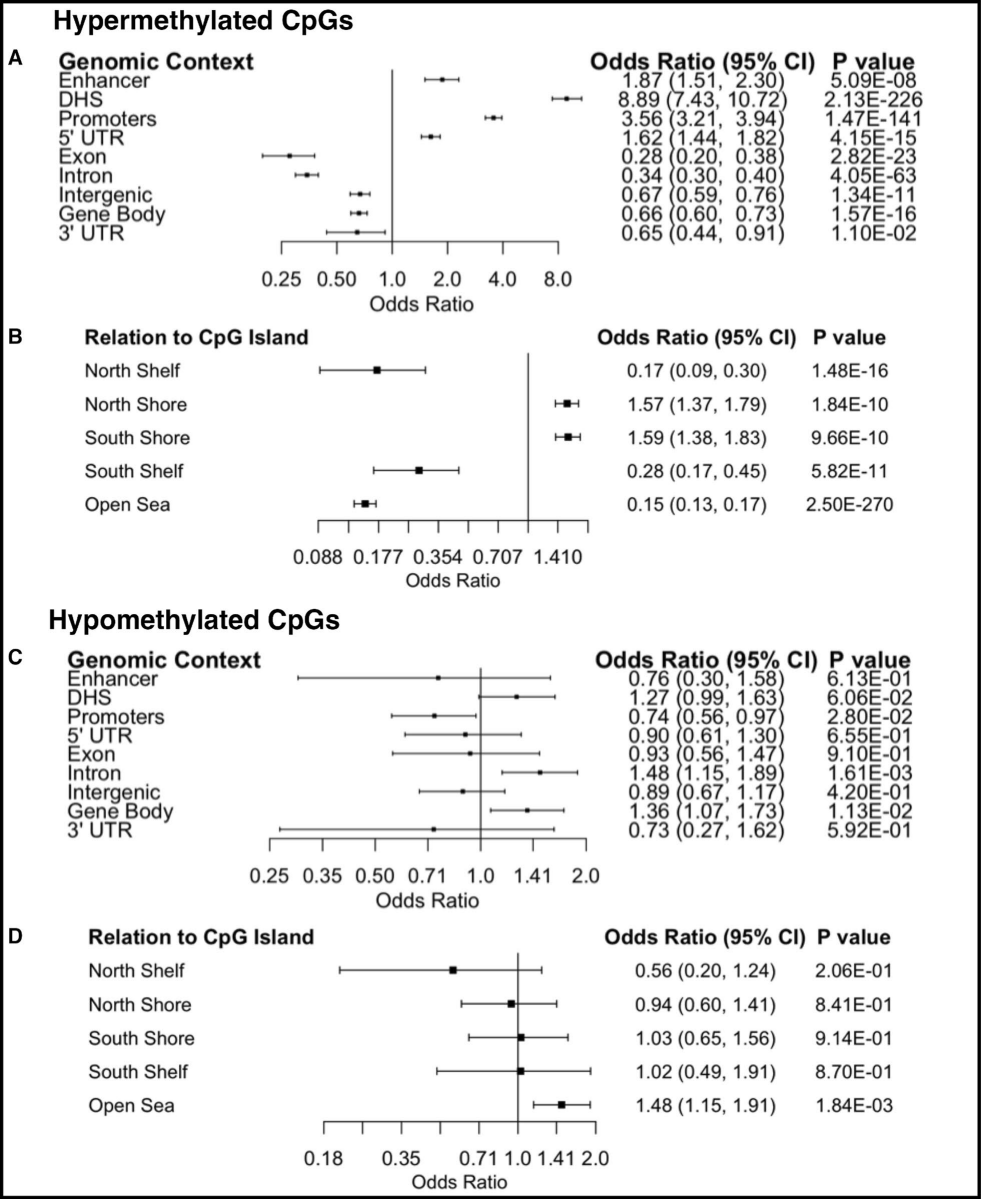
Genomic context and relation to CpG islands of hyper and hypomethylated CpGs associated with PCa

CpGs identified in the models correcting for cell-type proportions of the TME (*n* = 2093, Fig. 2) were used for downstream analyses. We first tested for potential enrichment in genomic context regions of CpGs with hypermethylation in prostate tumors and observed enrichment for enhancer regions (OR = 1.87, *P* = 5.09 × 10^–8^), DNase I hypersensitive sites (DHS) (OR = 8.89, *P* = 2.13 × 10^–226^), promoters (OR = 3.56, *P* = 1.47 × 10^–141^), and 5’ untranslated regions (OR = 1.62, *P* = 4.15 × 10^–15^), and depleted in exons (OR = 0.28, *P* = 2.82 × 10^–23^), introns (OR = 0.34, *P* = 4.05 × 10^–63^), intergenic regions (OR = 0.67, *P* = 1.34 × 10^–11^), gene bodies (OR = 0.66, *P* = 1.57 × 10^–16^), and 3’ untranslated regions (OR = 0.65, *P* = 1.10^–2^) (Fig. 3A). When hypermethylated CpGs associated with prostate cancer were assessed for genomic context enrichment relative to CpG islands, they were enriched for north and south shore regions (OR = 1.57, *P* = 1.84 × 10^–10^ and OR = 1.59, *P* = 9.66 × 10^–10^, respectively), and depleted in north and south shelves (OR = 0.17, *P* = 1.48 × 10^–16^ and OR = 0.28, *P* = 5.82 × 10^–11^, respectively) and open sea regions (OR = 0.15, *P* = 2.50 × 10^–270^) (Fig. 3B). Next, testing for genomic context enrichment analysis among CpGs hypomethylated in prostate cancer, we observed enrichment for gene bodies (OR = 1.36, *P* = 1.13 × 10^–2^) and introns (OR = 1.48, *P* = 1.61 × 10^–3^), and depletion among promoters (OR = 0.74, *P* = 2.80 × 10^–2^, Fig. 3C). When hypomethylated CpGs associated with prostate cancer were assessed for genomic context enrichment relative to CpG islands, they were enriched for open sea regions (OR = 1.48, *P* = 1.84 × 10^–3^, Fig. 3D). Of the top twenty most significant hypermethylated CpGs, eighteen out of twenty tracked to a gene (Additional file 2: Table S1). Only fourteen of the twenty most significant hypomethylated CpGs were tracked to a gene (Additional file 3: Table S2).

**Fig. 4:**
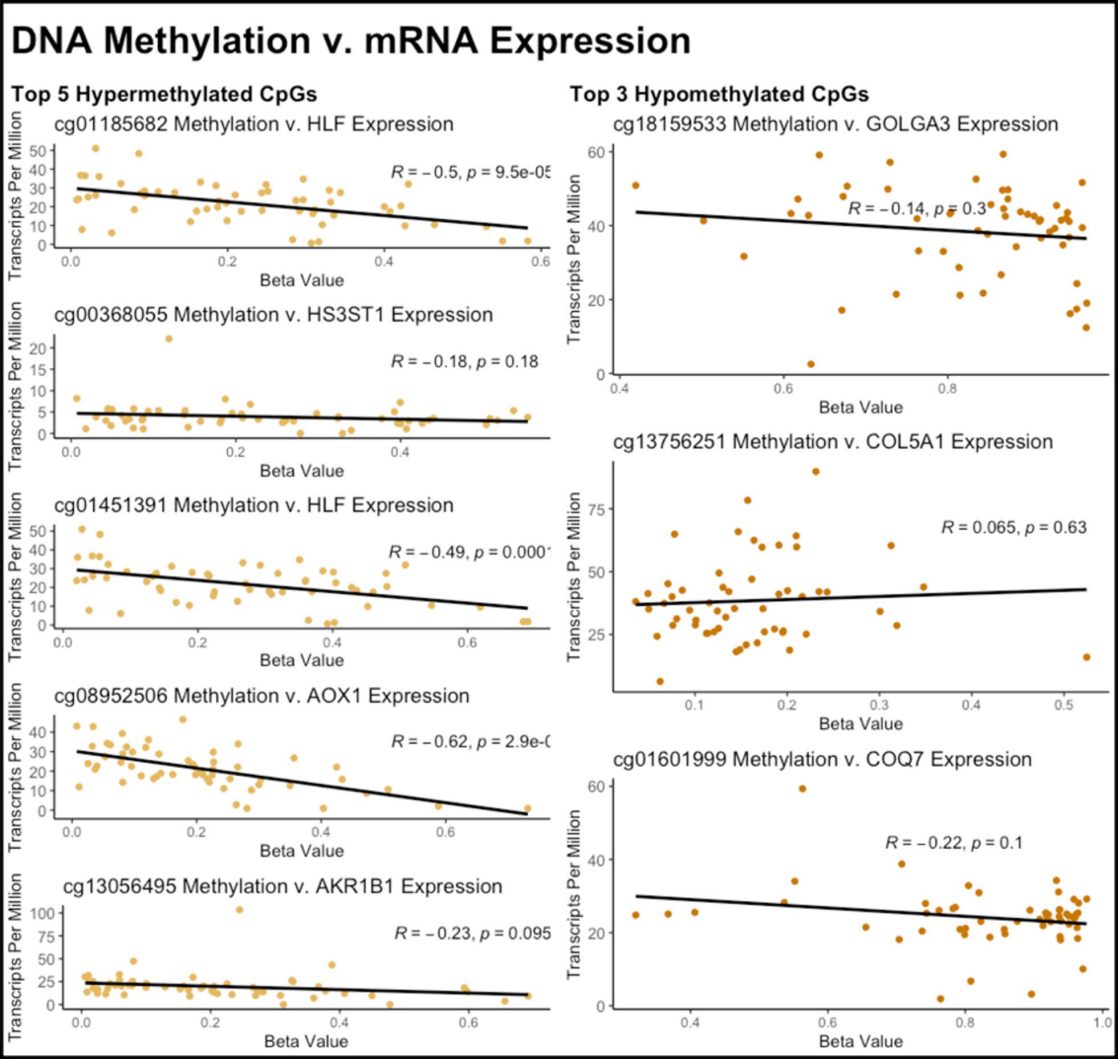
DNA methylation v. mRNA expression for the top hypermethylated and hypomethylated CpGs associated with PCa

When comparing the methylation levels of the top hypermethylated DMCs to the expression levels of the genes they mapped to (Fig. 4), a significant decrease in gene expression was found with an increase in methylation of cg01185682 (Gene = *HLF*, *P*-value = 9.5 × 10^–5^), cg01451391 (Gene = *HLF*, *P*-value = 1.4 × 10^–4^), and cg08952506 (Gene = *AOX1*, *P*-value = 2.9 × 10^–7^). None of the hypomethylated DMCs had a significant relationship between DNA methylation levels and gene expression (Fig. 4).

**Fig. 5:**
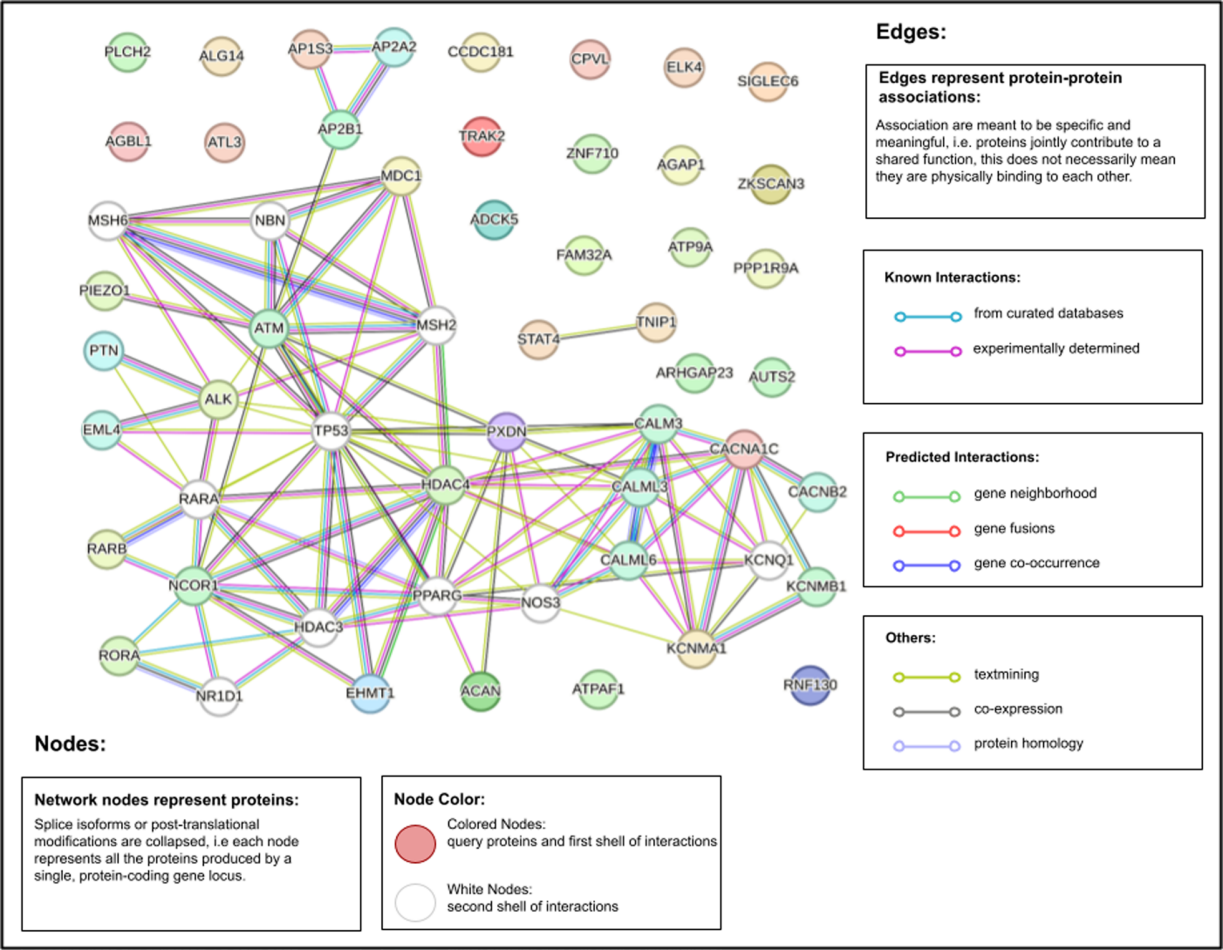
Induced network of the top twenty differentially methylated hyper and hypomethylated genes

The 2,093 CpGs with significantly altered methylation in prostate tumors from the fully adjusted EWAS mapped to 717 distinct genes. The top twenty hypermethylated genes with the largest number of associated CpGs are listed in Additional file 4: Table S3. The top five hypermethylated genes are *CCDC181* (10 CpGs), *CPVL* (9 CpGs), *GPR84-AS1* (9 CpGs), *ALK* (8 CpGs), and *LINC01929* (8 CpGs). The top twenty hypomethylated genes are listed in Additional file 5: Table S4. The top five hypomethylated genes are *AGAP1* (3 CpGs), *EHMT1* (2 CpGs), *ELK4* (2 CpGs), *FAM23OD* (2 CpGs), *HDAC4* (2 CpGs). All genes from Additional file 4: Tables S3, Additional file 5: Table S4 were used to build a functional protein association network (Fig. 5).

**Fig. 6:**
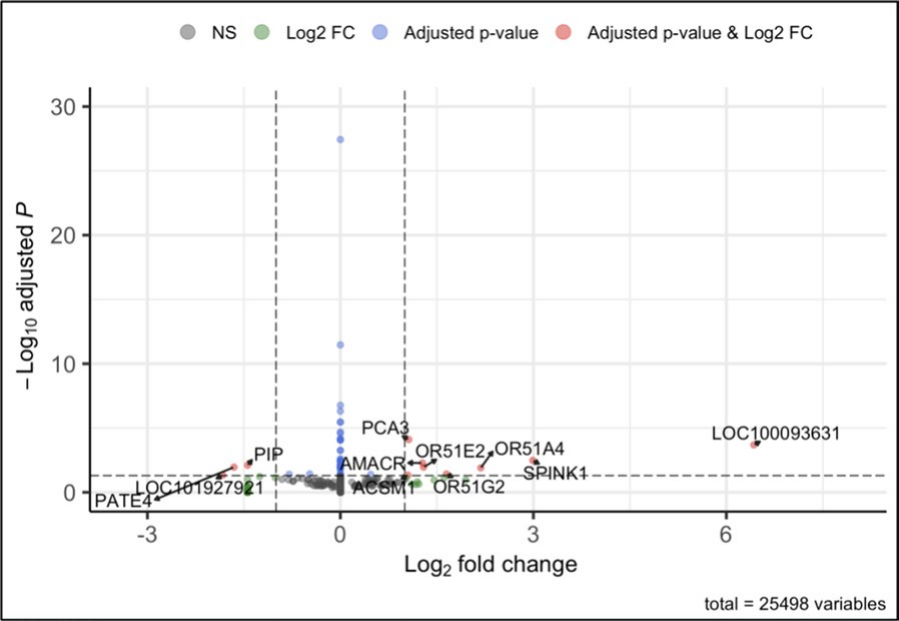
TME correction in DEA identifies 51 tumor-specific genes when comparing tumor versus non-tumor groups. The differential expression analysis pathological stage and for age, sex, Gleason score, preoperative PSA levels, pathological stage, and immune and angiogenic cell-type proportions from the second HiTIMED hierarchical level. Genes with significant adjusted *p*-values (FDR *Q*-value < 0.05) are shown in blue, those with log2 fold change (log2(tumor expression/control expression)) whose absolute value is greater than one in green, and genes that are both are shown in red

Two differential expression analyses were performed using paired tumor-healthy prostate tissue samples from 56 patients. The first was adjusted for age, race, Gleason score, pathological stage, and preoperative PSA levels (Additional file 1: Fig. S2A) and resulted in 3367 significantly differentially expressed genes (FDR *Q*-value < 0.05), associated with prostate cancer. The second was adjusted for age, race, Gleason score, pathological stage, preoperative PSA levels, the immune and angiogenic cell-type proportions from HiTIMED hierarchical level two (Additional file 1: Fig. S2B, Fig. 6) and identified 51 significantly differentially expressed genes (FDR *Q*-value < 0.05). Adding immune and angiogenic cell-type proportions identified a more tumor-specific set of genes when comparing the two differential expression analyses. Of the 51 genes that were significant after correcting for multiple comparisons, only ten had log2 fold changes whose absolute values were greater than one, meaning that the expression of a gene in the tumor tissue is either greater than or equal to double that of the healthy tissue or less than or equal to half of the control expression (Additional file 7: Table S6).

When comparing the genes found to be associated with prostate cancer from the epigenome-wide association study with those from the differential expression analysis, only two genes were found to be significant in both analyses: *SHISA3* and *LMX1A*. Both were slightly underexpressed compared to healthy prostate tissue and contained two CpG sites. The CpG sites for *SHISA3* were both hypermethylated, while one for *LMX1A* was hypermethylated, and one was hypomethylated.

**Fig. 7:**
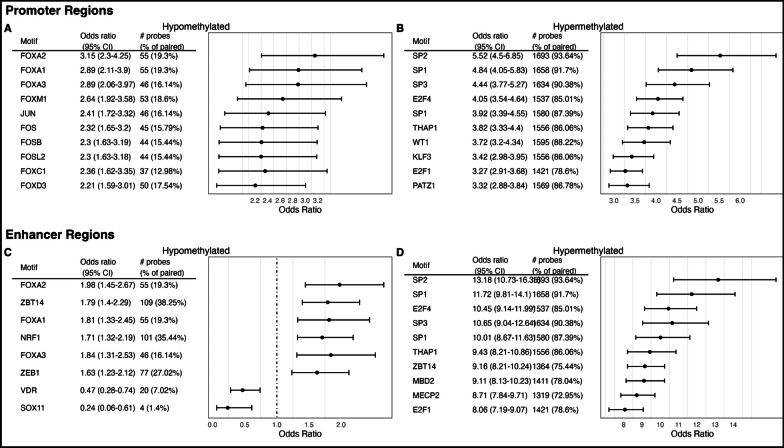
Transcription factor (TF) motifs associated with prostate cancer. TF motifs that are hypomethylated and in promoter regions (**A**), hypermethylated and in promoter regions (**B**), hypomethylated and in enhancer regions (**C**) and hypermethylated and in enhancer regions (**D**)

The 2,093 DMCs from the fully adjusted EWAS were utilized in an ELMER analysis to investigate the regulatory element and TF networks of prostate cancer. 193 hypomethylated promoter motifs were significantly associated with prostate cancer (Additional file 8: Table S7) and the top three were FOXA2 (OR = 3.15, FDR *Q*-value = 6.6 × 10^–10^), FOXA1 (OR = 2.89, FDR *Q*-value = 9.1 × 10^–9^), and FOXA3(OR = 2.89, FDR *Q*-value = 1.2 × 10^–7^) (Fig. 7A). 546 hypermethylated promoter motifs were significantly associated with prostate cancer (Additional file 9: Table S8) and the top three were SP2 (OR = 5.52, FDR *Q*-value = 6.3 × 10^–98^), SP1 (OR = 4.84, FDR *Q*-value = 8.6 × 10^–103^), and SP3 (OR = 4.44, FDR *Q*-value = 1.8 × 10^–102^) (Fig. 7B). 8 hypomethylated enhancer motifs were associated with prostate cancer (Additional file 10: Table S9) and the top three were FOXA2 (OR = 1.98, FDR *Q*-value = 7.8 × 10^–3^), ZBT14 (OR = 1.79, FDR *Q*-value = 2.6 × 10^–3^), and FOXA1 (OR = 1.81, FDR *Q*-value = 3.7 × 10^–2^) (Fig. 7C). 666 hypermethylated enhancer motifs were associated with prostate cancer (Additional file 11: Table S10) and the top three were SP2 (OR = 13.18, FDR *Q*-value = 5.4 × 10^–286^), SP1 (OR = 11.72, FDR *Q*-value = 3.1 × 10^–312^), and E2F4 (OR = 10.45, FDR *Q*-value < 2.2 × 10^308^) (Fig. 7D). Master regulator TFs (MRTFs) for the significantly associated motifs were identified. The top three MRTFs for FOXA2, FOXA1, and FOXA3—the top three hypomethylated promoter motifs—were DLX1, SIM2, and FEV (Additional file 1: Fig. S3A). The top three MRTFs for SP2, SP1, and SP3—the top three hypermethylated promoter motifs—were GLIS3, BNC2, and L3MBTL4 (Additional file 1: Fig. S3B). The top three MRTFs for FOXA2, ZBT14, and FOXA1—the top three hypermethylated enhancer motifs—were DLX1, SIM2, and FEV (Additional file 1: Fig. S3C). The top three MRTFs for SP2, SP1, and E2F4—the top three hypermethylated enhancer motifs—were GLIS3, BNC2, and L3MBTL4 (Additional file 1: Fig. S3D).

## Discussion

This study aimed to identify the key epigenetic and gene expression alterations associated with prostate carcinogenesis, including adjustment for variation in cell types in the TME. To investigate the epigenome, differential methylation of CpG sites, either hyper or hypomethylated, was assessed, including tracking to genes. In addition, transcriptome alterations were assessed by comparing mRNA counts between the healthy and tumorous prostate tissue. Using correction for cell-type proportions in the TME in the epigenome-wide association study and the differential expression analysis allowed the identification of more disease-specific alterations.

Although all cells in an individual contain the same genome, different cell types have distinct phenotypes and functions. Epigenetic alterations, such as DNA methylation, regulate gene expression and shape the phenotypes and functions of different cell types, and therefore, each cell type has a distinct DNA methylation pattern [14]. Therefore, without correcting for cell type when identifying DMCs between cases and controls, variation in cell-type proportions among samples can confound associations of methylation identified using EWAS.

The top five most significant hypermethylated DMCs mapped to *HLF*, *HS3ST1*, *AOX1*, *AKR1B1*. *HLF*, hepatic leukemia factor, is a TF that has been found to promote ferroptosis resistance and encourage cell proliferation in triple-negative breast cancer [15]. Although DNA methylation in the gene body has sometimes been found to be associated with increased transcription [16] and two of the top five hypermethylated DMCs were both located in the gene body of *HLF*, increased methylation in both of the DMCs in *HLF* was associated with decreased expression of *HLF*. This inverse association might suggest a potential alternative transcriptional regulation of HLF in prostate cancer instead. *HS3ST1* has been found to promote tumor non-small-cell lung cancer progression by regulating SPOP/FADD/NF-κB pathway [17]. The DMC located in the *HS3ST1* gene is located in the 5' untranslated region, which could potentially prevent the binding of TFs and alter its regulation. Although *AKR1B1* has also been found to be less expressed in prostate tumors [18], its role in prostate cancer remains unknown. It, however, has been found to be associated with tumor progression and is overexpressed in colorectal, breast, ovarian, cervical, rectal, pancreatic, and lung cancers while being underexpressed in endometrial and adrenocortical cancers [19]. *AOX1* encodes a protein that produces hydrogen peroxide and, under certain conditions, can catalyze the formation of superoxide. Our results align with recent studies that have found *AOX1* to be hypermethylated in prostate cancer [20]; however, the effect of decreased *AOX1* expression in prostate cancer has not been studied. In advanced bladder cancer, low levels of *AOX1* in normal bladder epithelial cells lead to the rewiring of the tryptophan-kynurenine pathway, resulting in elevated NADP levels, which could increase metabolic flux through the pentose phosphate (PPP) pathway and enable increased nucleotide synthesis, therefore promoting cell invasion during advanced bladder cancer progression [21]. Further studies would be needed to see if *AOX1* acts similarly in prostate cancer.

The top five most significant hypomethylated DMCs mapped to *GOLGA3*, *COL5A1*, and *COQ7*. Of the hypomethylated genes, *GOLPH3* promotes cell proliferation by enhancing the activity of AKT-mTOR signaling and plays a key role in tumor proliferation and cell cycle regulation of prostate cancer [22]. The role of *COL5A1*, which encodes a component of type V collagen, in prostate cancer remains unknown. However, its overexpression promotes clear cell renal cell carcinoma (CRCC), and its knockdown in in vitro CRCC cell lines induced cell apoptosis and inhibited cell migration and invasion [23]. *COQ7* is involved in ubiquinone biosynthesis, but its role in prostate cancer has not been studied yet. Therefore, all three of these genes could be potentially good candidates for further study, especially *GOLPH3* and *COL5A1*, whose expressions were found to be associated with tumor progression in other tumor types.

The list of DMCs was then used to find genetic pathways associated with prostate cancer. These were found by conducting a gene ontology (GO) term analysis, which looks at which classes of genes are overrepresented in a large group of genes. The three most significant pathways found were system development, multicellular organism development, and regulation of cell differentiation (Additional file 6: Table S5)﻿.

While investigating the individual genes found to be associated with prostate cancer, the three genes with the largest number of hypermethylated DMCs contained in them are *CCDC181*, *CPVL*, and *GPR84-AS1*. These genes encode the following proteins: coiled-coil domain-containing protein 181, carboxypeptidase vitellogenic-like protein, and G-protein coupled receptor 84 antisense RNA 1, respectively. Coiled-coil domain-containing protein 181 is a microtubule-binding protein involved in spermatogenesis [24]. Its function in prostate cancer is unknown, but it has been found to be hypermethylated and underexpressed in prostate tumors [25]. Carboxypeptidase vitellogenic-like protein is a novel carboxypeptidase—which cleaves single amino acids in the carboxyl termini of proteins—whose exact function is unknown [26]. Increased levels of carboxypeptidase vitellogenic-like protein have been found in prostate tumors, but their contributions to tumor progression have not been identified [27]. GPR84-AS1 is an antisense RNA that binds to the G-protein coupled receptor 84, which is a part of the G protein-coupled receptor family whose activation is involved in the inflammatory response [28] and blocks its translation into protein. While found to be upregulated in breast cancer [29], the function and regulation of GPR84-AS1 in prostate cancer have not been studied before.

The three genes with the largest number of hypomethylated DMCs contained in them are *AGAP1*, *EHMT1*, and *ELK4*. These genes encode the following proteins: ArfGAP with GTPase domain, ankyrin repeat, and PH domain 1; euchromatic histone lysine methyltransferase 1; and ETS transcription factor ELK4, respectively. While *AGAP1* is found to be downregulated in osteosarcoma [30], its expression levels and function in prostate cancer have not been investigated. *EHMT1*'s expression in prostate cancer has not been studied; however, it is overexpressed in lung cancer and cells that underwent *EHMT1* knockdown-induced apoptosis and G1 cell cycle arrest [31]. *ELK4* is involved in cell growth promotion and is found to be overexpressed in prostate cancer [32].

A functional protein association network was built using the top twenty hyper and hypomethylated genes as input to *STRING* [33], which accesses the BIND, DIP, GRID, HPRD, IntAct, MINT, and PID databases for its experimental protein–protein interaction data and Biocarta, BioCyc, GO, KEGG, and Reactome for its curated protein–protein interaction data. The resulting network was composed of numerous tumor-related genes centered on the protooncogene TP53. In the surrounding first layer and nodes connected to TP53, there were histone deacetylases (HDAC4, HDAC3), transcription factors (RARA), DNA repair proteins (MSH2, MSH6, NBN, ATM), and cell cycle checkpoint proteins (MDC1).

For our differential expression analysis (DEA), while previous studies had investigated the transcriptome of prostate cancer, this study included correction for cell-type proportions in the TME aiming to discern more tumor-specific altered gene expression. This was done by comparing a DEA that corrected for TME cell-type proportions with one that did not. In the DEA that did not correct for TME cell-type proportions, a much larger scope of differentially expressed genes (DEG) associated with prostate cancer was identified, indicating that variation in cell-type proportions across samples is an important potential confounder of differential gene expression analysis in prostate cancer. Just as in the EWAS, utilizing *HiTIMED* TME deconvolution to reduce the number of genes whose expression was associated with prostate cancer resulted in a more succinct and potentially disease-specific list. The top three overexpressed genes whose differential expression was associated with prostate cancer are *PCA3*, *SPINK1*, and *AMACR,* which encode prostate cancer antigen 3, serine protease inhibitor Kazal-type 1, alpha-methylacyl-CoA racemase. *PCA3* is a well-known overexpressed gene in prostate cancer and has been identified as a possible candidate as an early detection biomarker. Because elevated PSA levels can be due to several factors other than prostate cancer, *PCA3* would offer more specificity when determining the presence of a tumor in the prostate [34]. However, little is known about the role of *PCA3* in prostate cancer, except that PCA3 silencing decreases cell growth and survival and induces apoptotic cell death [35]. *SPINK1* has been found to stimulate cell proliferation and contributes to prostate cancer cell plasticity through its interaction with the epidermal growth factor receptor [36]. *AMACR* is known to be overexpressed in prostate cancer and is thought to metabolically support tumor growth through β-oxidation of certain fatty acids, as well as damage DNA through peroxide production [37].

*PATE4* is the only underexpressed gene from the differential expression analysis to be significant and have a log2 fold change less than negative one. *PATE4*, which encodes prostate and testis expressed 4, has been found to be underexpressed in prostate cancer [7]; however, how it contributes to prostate cancer remains unknown.

Examining TF regulatory networks, FOXA1 and FOXA2 promoter and enhancer regions were hypomethylated when compared to non-tumor control tissue. FOXA1 and FOXA2 have been found to drive lineage plasticity in neuroendocrine prostate cancer, especially in response to androgen deprivation treatment [38, 39]. We also found SP family and E2F4 enhancer and promoter regions to be hypermethylated. The SP family is known to be overexpressed in numerous cancers [40], including prostate cancer [41], suggesting potential alternative methylomic regulation of these promoter and enhancer regions. E2F4 is known to repress *Bub3* and *Pttg1*, two important mitotic genes, in prostate cancer and play a crucial role in G_2_ arrest [42]. Therefore, down-regulation of E2F4 in prostate cancer could lead to an increase in mutations and tumor progression and proliferation.

There are some limitations to our approach. The deconvolutional tool *HiTIMED* was trained on tumor data, not healthy prostate data. Therefore, the cell-type proportion results will be more accurate for the cancerous samples. Additionally, as seen in Fig. 1, *HiTIMED* predicts a small percentage of tumor cells in healthy prostate tissue samples. This is because, as of right now, the *HiTIMED* model forces some percentage of cells to be classified as tumor cells, and in the adjacent histopathological nontumor prostate cells, there might be some preneoplastic cells with epigenetic patterns similar to the tumor cells that will therefore be classified as tumor cells. An improved *HiTIMED* deconvolution model that allows for healthy tissue deconvolution could improve the results; however, it is still evident from Additional file 1: Fig. S1, and Additional file 1: Fig. S2 that correcting for cell types of the TME leads to more tumor-specific results in epigenome-wide association and differential expression analyses. Further study to validate these results could be performed using ESTIMATE [43], an expression-based deconvolution model that also predicts tumor, immune, and stromal proportions. Additionally, while *HiTIMED* predicts 17 different cell types of the TME, cell-state resolution, such as separating cancer-associated fibroblasts from the stroma cell-type group (which is a part of the angiogenic cell-type category used in our corrections), could help better elucidate the biology of prostate cancer. DMCs associated with CAFs in prostate cancer could be used to expand *HiTIMED* deconvolution in prostate cancer [44].

## Conclusions

This study identifies a more disease-specific list of genes and epigenetic markers associated with prostate cancer. We implemented a novel epigenome-wide association study and differential expression approach that identified a more tumor-cell-specific set of altered genes and epigenetic marks establishing a more confounding-adverse methodology and illustrating the benefits of correcting for cell types of the TME when performing these studies. These genes and epigenetic sites can be further studied to identify their individual contributions to PCa tumorigenesis and tumor progression. Understanding the contributions of the gene regulation networks acting on PCa tumors will allow physicians to better treat their patients, leading to improved patient outcomes. Additionally, these genes and epigenetic sites can be further investigated as potential biomarkers of disease and therapeutic targets.

## Methods

### Study population

The data for this study came from Gene Expression Omnibus's GSE183040 dataset. The data was collected from fifty-eight patients with prostate cancer with a mean age of 62.8 years. After initial quality control of the DNA methylation data, two patients were removed, and the final dataset had fifty-six patients with a mean age of 63.1 years. Each patient underwent a radical prostatectomy before any treatment, where tumor and healthy prostate tissues were collected, creating case–control paired samples. In addition to the prostate tissue samples, data was gathered on patient age, race, preoperative PSA levels, pathological stage, and Gleason scores. PSA is an enzyme produced by prostate cells, and elevated levels are associated with prostate cancer. Preoperative PSA levels ranged from 1.2 to 13.0 ng/ml of blood. The pathological stage measures the tumor's size and location, and samples range from T2a to T3b. Lastly, the Gleason Score is used to grade the composition of the tumor and how it compares to the healthy prostate tissue. The Gleason Score is composed of two to three scores based on the tumor's primary, secondary, and sometimes tertiary patterns. Patterns that resemble normal, healthy prostate tissue receive low scores, while cancerous patterns receive higher scores. Samples ranged from *3* + *3 T4* to *5* + *5 T4*. See Table 1 for more information on the study population. Covariates such as preoperative PSA levels, pathological scores, and Gleason scores were compared between patients who experienced a recurrence of their tumor versus those who experienced remission.

### Data collection

The DNA methylation and RNA-seq data used in this study were downloaded from the publicly available dataset GSE183040 (https://www.ncbi.nlm.nih.gov/geo/query/acc.cgi?acc=GSE183040) and are available at the National Center for Biotechnology’s Gene Expression Omnibus repository.

### Quality control and data preprocessing

Quality control was performed using *ENmix’s* [45] *qcinfo* function. Two subjects were identified as having low quality samples, and their samples were removed. IDAT files were preprocessed using *ENmix’s mpreprocess* function. This function performs background correction, dye bias correction, inter-array normalization and probe-type bias correction. Additionally, masked CpGs—CpGs that are cross-reactive, SNP-associated, on sex chromosomes, or are sites of non-CpG (CpH) methylation – were removed.

### Tumor microenvironment deconvolution

Estimation of the cell types of the TME was performed using the *HiTIMED_deconvolution* function from the *HTIMED* R package [13]. *HiTIMED* deconvolution is equivalent to *InfiniumPurify* [6] at hierarchical level one and deconvolves the TME at increasing resolution in layers two through six. The TME was deconvolved at hierarchical layer six and two. Layer six estimates monocytes, basophils, eosinophils, neutrophils, naïve B, memory B, CD4 naïve T, CD4 memory T, regulatory T, CD8 naïve T, CD8 memory T, dendritic cell, natural killer, endothelial, epithelial, stromal, and tumor cell-type proportions. Layer two estimates tumor, angiogenic (composed of endothelial, epithelial, and stromal cells), and immune (composed of neutrophil, basophil, eosinophil, monocyte, dendritic, natural killer, naïve B, memory B, naïve CD4T, memory CD4T, regulatory T, naïve CD8T, and memory CD8T cells) cell-type proportions. More information regarding the cell types of other layers of *HiTIMED* can be found in Fig. 1 of the Zhang et al. (2022) paper. Tumor, angiogenic, and immune cell-type proportions from hierarchical level two were then compared between patients who experienced a recurrence of their tumor versus those who experienced remission.

### Epigenome-wide association study

An epigenome-wide association study (EWAS) was used to identify differentially methylated CpG sites between the healthy prostate and cancerous tissue. M-values, a measurement of methylation, were regressed linearly on disease state (i.e., tumor v. control) with adjustment for certain covariates. There were three EWAS studies performed in this study. The first was adjusted for age and race and blocked on patient ID. The second was adjusted for age, race, Gleason score, pathological stage, preoperative PSA levels, and blocked on patient ID. The third was adjusted for age, race, Gleason score, pathological stage, preoperative PSA levels, and immune and angiogenic cell-type proportions (from the HiTIMED second hierarchical deconvolution layer) and blocked on patient ID. Due to low counts in certain pathological stages and Gleason score groups, these covariates were grouped for the regression. Gleason score was grouped into primary tumor patterns of three as one group and four and five combined as the other. The pathological stage was grouped into stage two and stage three. The EWAS was performed using the *lmFit* function from the R package *limma* [46]. Multiple comparisons were corrected for using a false discovery rate of five percent. The EWASs were performed on 56 prostate cancer patients, each with both a healthy and cancerous prostate tissue sample.

### Downstream analyses

#### Genetic analysis

A genetic analysis of the CpG sites found to be associated with prostate cancer from the EWAS was performed to map the CpGs to specific genes. The *biomaRt* R package [47] was used to query the Ensembl Homo Sapiens Database and determine the genomic location of each associated CpG. If the CpG was located on a gene, the HUGO Gene Nomenclature Committee (HGNC) symbol of the gene and the total number of associated CpGs located on each gene was recorded.

#### Gene ontology (GO) term analysis

A GO term analysis was performed on the CpG sites found to be associated with prostate cancer from the EWAS using the *gometh* function from the *missMethyl* R package [48]. This function takes into account the number of CpG probes per gene in addition to CpGs that map to numerous genes to return significant GO terms: a term that represents a biological process, molecular function, or cellular component that shares a genetic pathway.

#### Genomic context

Genomic-context enrichment analysis was performed on the differentially methylated CpG sites using the *IlluminaHumanMethylationEPICanno.ilm10b4.hg19* annotation file [49]. Odds ratios of the differentially methylated CpG compared with total CpG probes measured were calculated for the north shelf, north shore, south shelf, south shore, and open sea regions surrounding the CpG islands and genomic regions such as enhancers, DNase I hypersensitive sites (DHS), promoters, 5' untranslated regions (UTR), exons, introns, intergenic regions, gene bodies, and 3' UTRs. Additionally, p-values were calculated using the *Fisher's Exact Test* to see if these odds ratios were significantly different than one (which would signify that the prostate cancer-associated CpG sites are equally likely as the non-associated CpG sites to be found in that region).

### Functional protein association network

*STRING* (https://string-db.org/) to create the functional protein association network [33]. *STRING* uses the BIND, DIP, GRID, HPRD, IntAct, MINT, and PID databases for its experimental data and Biocarta, BioCyc, GO, KEGG, and Reactome for its curated data. The nodes of the network represent different proteins and the edges represent either known or predicted interactions. The top twenty hypermethylated and top twenty hypermethylated genes from Additional file 4: Tables S3 and Additional file 5: Table S4 were used as inputs to create the network in Fig. 5.

### Differential expression analysis

The differential expression analysis was performed using the *DESeq2* R package [50]. There were two differential expression analyses performed. The first adjusted for age, sex, Gleason score, preoperative PSA Level, and pathological stage, while the second adjusted for the covariates previously stated in addition to immune and angiogenic cell-type proportions (from the HiTIMED second hierarchical deconvolution layer). The RNA-seq data were normalized to control for differences in sequencing depth, which can differ between samples, and RNA composition, which can skew differential expression analyses. The variance of gene counts was estimated and shrunk for the genes with lower counts so that significant changes could be detected in genes with low replicate counts. Lastly, the *lfcShrink*function from *DESeq2* was used to downweight genes with a high log2-fold changes so that the fold changes were not due to low counts before contrasting the tumor versus normal groups. The package *EnhancedVolcano* was used to create the volcano plots [51].

### ELMER (Enhancer linking by methylation/expression relationships) analysis

The ELMER analysis was performed using the *ELMER* R package [52]. The *get.pair* function was used on the 2,093 DMCs identified from the fully adjusted EWAS to link altered methylation of distal probes to target genes with altered expression levels in prostate cancer. Next, the *get.enriched.motif* function was used to identify motifs from the HOCOMOCO (HOmo sapiens COmprehensive MOdel COllection) v11 using the HOMER algorithm that are either 250 bp up or downstream of the probes in these significant probe-gene pairs. Lastly, the *get.TFs* function is utilized to identify master regulator TFs that bind to the previously identified motifs.

### Supplementary Information

**Additional file 1.** **Fig. S1** TME correction in EWAS identifies more tumor-specific CpGs when comparing tumor versus non-tumor groups. Epigenome-wide association study of prostate cancer and matched non-tumor normal prostate tissue adjusted for (**A**) patient age and race, and blocked on patient ID, (**B**) patient age, race, Gleason score, pathological stage, preoperative PSA levels, and blocked on patient ID, (**C**) patient age, race, Gleason score, pathological stage, and preoperative PSA levels, immune and angiogenic cell-type proportions from HiTIMED hierarchical level two, and blocked on patient ID. Each point represents a CpG site; in total, 746,980 CpG sites are shown, and those with an FDR *Q*-value < 0.05 are shown in red (2093 CpGs). FDR *Q*-value < 0.05 is shown above the blue line, and FDR *Q*-value < 0.01 is shown above the red line; **Fig. S2** TME correction in DEAs identifies more tumor-specific CpGs when comparing tumor versus non-tumor groups. Associated genes of prostate cancer were reduced from 3,367 in panel **A** to 51 in panel **B**. The differential expression analysis in panel **A** was corrected for age, sex, Gleason score, preoperative PSA levels, and pathological stage and for age, sex, Gleason score, preoperative PSA levels, pathological stage, and immune and angiogenic cell-type proportions from the second HiTIMED hierarchical level in panel *B*. Genes with significant adjusted *p*-values (FDR *Q*-value<0.05) are shown in blue, those with log2 fold change (log2(tumor expression/control expression)) whose absolute value is greater than one in green, and genes that are both are shown in red; **Fig. S3** Transcription factors associated with prostate cancer. TFs associated with the top three TF motifs for hypomethylated and in promoter regions (**A**), hypermethylated and in promoter regions (**B**), hypomethylated and in enhancer regions (**C**) and hypermethylated and in enhancer regions (**D**). In panels **A–D**, red denotes the top three most significant TFs, yellow denotes same family, and blue denotes same subfamily. *P*-values were corrected for using a false discovery rate of 0.05.

**Additional file 2**. **Table S1** Top twenty most significant hypermethylated CpGs associated with prostate cancer.

**Additional file 3**. **Table S2** Top twenty most significant hypomethylated CpGs associated with prostate cancer.

**Additional file 4**. **Table S3** Top twenty hypermethylated genes containing the most differentially methylated CpGs.

**Additional file 5**. **Table S4** Top twenty hypomethylated genes containing the most differentially methylated CpGs.

**Additional file 6**. **Table S5** Top twenty most significant GO terms.

**Additional file 7**. **Table S6** Genes associated with prostate cancer whose absolute value of their log2 fold change is greater than one.

**Additional file 8**. **Table S7**. Hypomethylated Promoter Motifs.

**Additional file 9**. **Table S8**. Hypermethylated Promoter Motifs.

**Additional file 10**. **Table S9**. Hypomethylated Enhancer Motifs.

**Additional file 11**. **Table S10**. Hypermethylated Enhancer Motifs.

## Data Availability

The DNA methylation and RNA-seq datasets analyzed during the current study are available under the National Center for Biotechnology’s Gene Expression Omnibus repository with the accession ID—GSE183040. https://www.ncbi.nlm.nih.gov/geo/query/acc.cgi?acc=GSE183040.

## Abbreviations

PSA: Prostate-specific antigen
CpG: A region of DNA where a cytosine is followed by a guanine that has the potential to be methylated
EWAS: Epigenome-wide association study
FDR: False discovery rate
HGNC: HUGO Gene Nomenclature Committee
GO: Gene ontology
DHS: DNase hypersensitive sites
UTR: Untranslated region
OR: Odds ratio
DMC: Differentially methylated CpG
DEA: Differential expression analysis
DEG: Differentially expressed gene
TF: Transcription factor
MRTF: Master regulator transcription factor
