# Supplementary material for: Tumor microenvironment deconvolution identifies cell-type-independent aberrant DNA methylation and gene expression in prostate cancer
Source: Clin Epigenetics. 2024 Jan 3;16:5. doi: 10.1186/s13148-023-01609-3 (PMC10765773; doi:10.1186/s13148-023-01609-3)
Supplement: Supplementary file 1 — Additional file 1. Fig. S1 TME correction in EWAS identifies more tumor-specific CpGs when comparing tumor versus non-tumor groups. Epigenome-wide association study of prostate cancer and matched non-tumor normal prostate tissue adjusted for (A) patient age and race, and blocked on patient ID, (B) patient age, race, Gleason score, pathological stage, preoperative PSA levels, and blocked on patient ID, (C) patient age, race, Gleason score, pathological stage, and preoperative PSA levels, immune and angiogenic cell-type proportions from HiTIMED hierarchical level two, and blocked on patient ID. Each point represents a CpG site; in total, 746,980 CpG sites are shown, and those with an FDR Q-value < 0.05 are shown in red (2093 CpGs). FDR Q-value < 0.05 is shown above the blue line, and FDR Q-value < 0.01 is shown above the red line; Fig. S2 TME correction in DEAs identifies more tumor-specific CpGs when comparing tumor versus non-tumor groups. Associated genes of prostate cancer were reduced from 3,367 in panel A to 51 in panel B. The differential expression analysis in panel A was corrected for age, sex, Gleason score, preoperative PSA levels, and pathological stage and for age, sex, Gleason score, preoperative PSA levels, pathological stage, and immune and angiogenic cell-type proportions from the second HiTIMED hierarchical level in panel B. Genes with significant adjusted p-values (FDR Q-value<0.05) are shown in blue, those with log2 fold change (log2(tumor expression/control expression)) whose absolute value is greater than one in green, and genes that are both are shown in red; Fig. S3 Transcription factors associated with prostate cancer. TFs associated with the top three TF motifs for hypomethylated and in promoter regions (A), hypermethylated and in promoter regions (B), hypomethylated and in enhancer regions (C) and hypermethylated and in enhancer regions (D). In panels A–D, red denotes the top three most significant TFs, yellow denotes same family, and blue d [file 13148_2023_1609_MOESM1_ESM.docx]

**Supplementary Figures**:


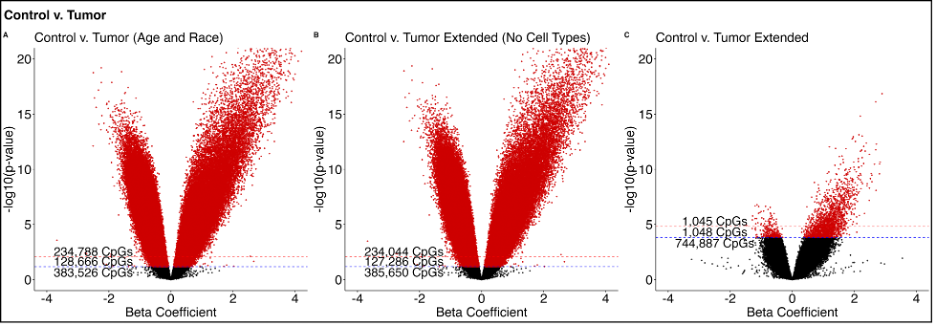


***Supplementary Figure 1*. TME correction in EWAS identifies more tumor-specific CpGs when comparing tumor versus non-tumor groups.** Epigenome-wide association study of prostate cancer and matched non-tumor normal prostate tissue adjusted for (A) patient age and race, and blocked on patient ID, (B) patient age, race, Gleason score, pathological stage, preoperative PSA levels, and blocked on patient ID, (C) patient age, race, Gleason score, pathological stage, and preoperative PSA levels, immune and angiogenic cell type proportions from HiTIMED hierarchical level two, and blocked on patient ID. Each point represents a CpG site; in total, 746,980 CpG sites are shown, and those with an FDR Q-value<0.05 are shown in red (2,093 CpGs). FDR Q-value<0.05 is shown above the blue line, and FDR Q-value<0.01 is shown above the red line.


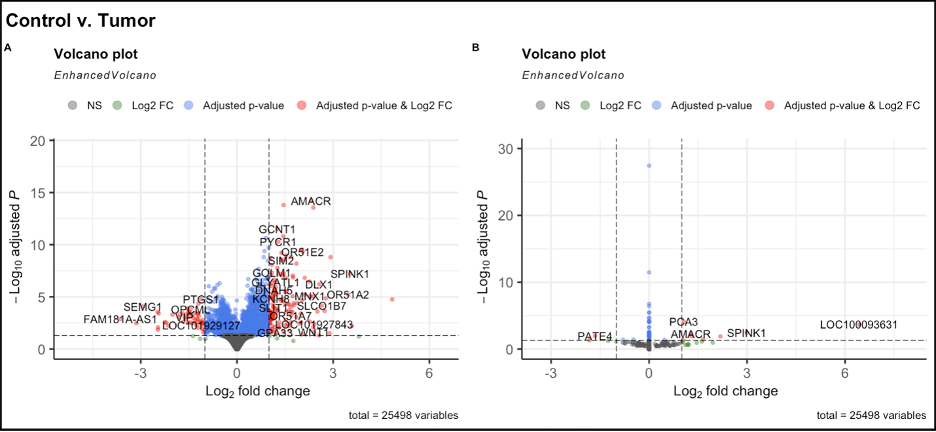


***Supplementary Figure 2*. TME correction in DEAs identifies more tumor-specific CpGs when comparing tumor versus non-tumor groups.** Associated genes of prostate cancer were reduced from 3,367 in panel A to 51 in panel B. The differential expression analysis in panel A was corrected for age, sex, Gleason score, preoperative PSA levels, and pathological stage and for age, sex, Gleason score, preoperative PSA levels, pathological stage, and immune and angiogenic cell type proportions from the second HiTIMED hierarchical level in panel B. Genes with significant adjusted p-values (FDR Q-value<0.05) are shown in blue, those with log2 fold change (log2(tumor expression/control expression)) whose absolute value is greater than one in green, and genes that are both are shown in red.


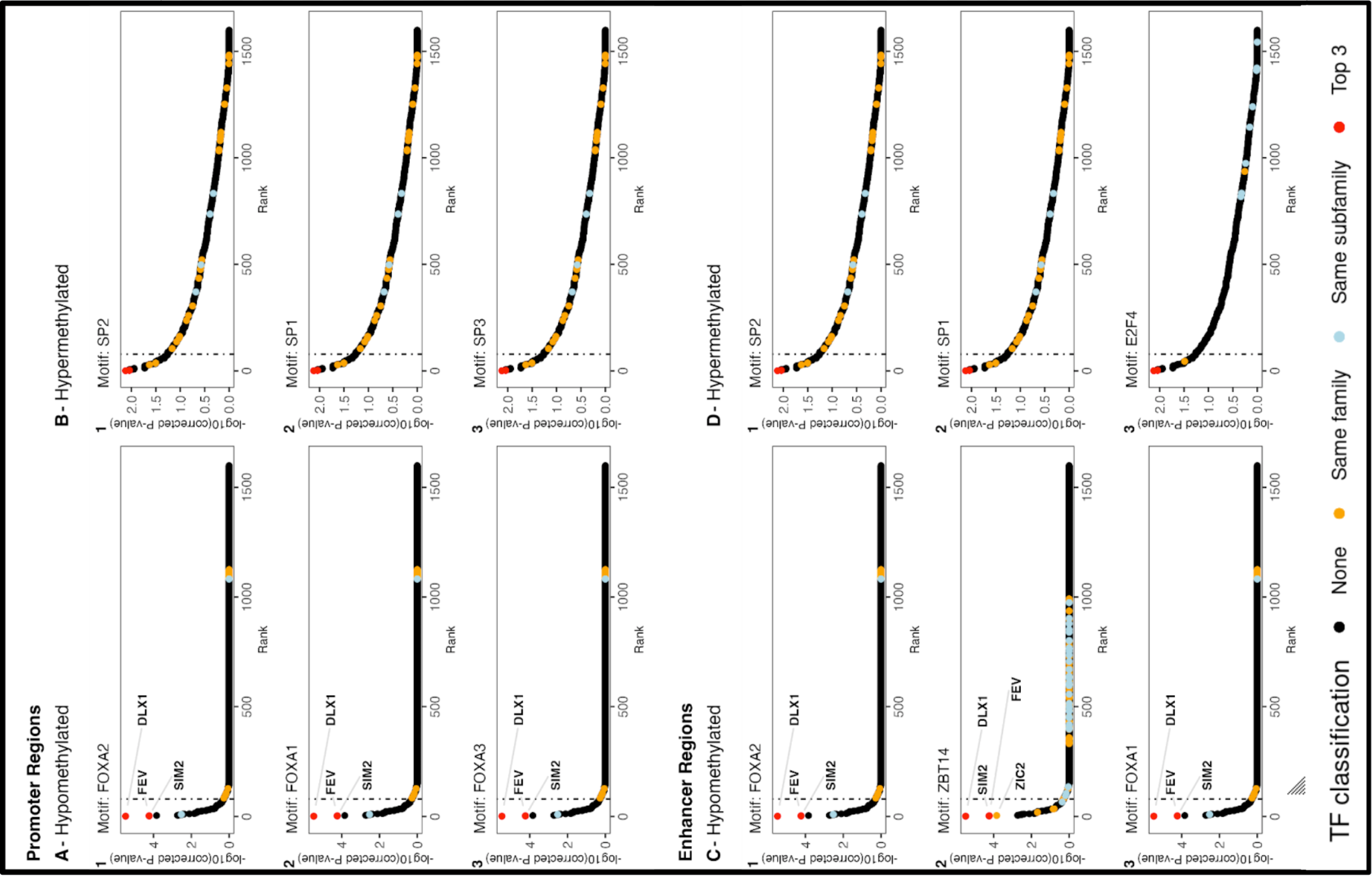


***Supplementary Figure 3.*  Transcription factors associated with prostate cancer.** TFs associated with the top three TF motifs for hypomethylated and in promoter regions (A), hypermethylated and in promoter regions (B), hypomethylated and in enhancer regions (C) and hypermethylated and in enhancer regions (D). In panels A, B, C, and D, red denotes the top three most significant TFs, yellow denotes same family, and blue denotes same subfamily. P-values were corrected for using a false discovery rate of 0.05.
